# The Safety of Chloroform

**Published:** 1904-09

**Authors:** 


### *EDITORIAL NOTES AND COMMENTS.*

The Business office of THE JOURNAL-RECORD is No. 1007-1008 Century Building.

The Editorial office is 318-319-320 The Prudential.

Address all Business communications to Dr. M. B. Hutchins, Mgr.

Make remittances payable to THE ATLANTA JOURNAL-RECORD OF MEDICINE.

On matters pertaining to the Editorial and Original Communications address Dr. Bernard Wolff, Atlanta.

Reprints of original articles will be furnished at cost price. Requests for the same should always be made *on the manuscript.*

We will present, post-paid, on request, to each contributor of an original article, twenty (20) marked copies of THE JOURNAL-RECORD containing such article.

### THE SAFETY OF CHLOROFORM.

IT may be said that, in the minds of the laity and to some extent the medical profession, the chief obstacle in the way of surgical operations is dread of the anesthetic. The number of deaths from chloroform narcosis (to put ether out of the discussion), when compared to the prevalence of the use of the drug, is insignificant; but deaths do occur, and often so suddenly and unexpectedly as to be charged against caprice rather than the dose of the drug. The large amount of investigation which has been undertaken on this subject has had at least one conspicuously practical effect—the anesthesia is no longer entrusted to the tyro, but those are selected to administer it who have had the necessary experience to guide them in this most important adjunct to surgical procedure. The third report of the special chloroform committee of the British Medical Association is the latest work on the subject of chloroform anesthesia, and deals principally with percentages as regards the dose. The gist of the report, although the committee's labors are not yet by any means completed, is that the effect of chloroform is in proportion to the dose, and that a small dose, properly administered with a suitable inhaler, even as low as two per cent. chloroform in atmospheric air, will produce surgical anesthesia with a minimum of risk. Experiments on the isolated heart brought out the fact that small doses exerted a depressant effect, but this effect disappeared in the withdrawal of the chloroform—while under large doses the depressant effect was permanent. It was also observed that the blood takes up and retains no small part of the chloroform, a circumstance involving a possible menace to safety, especially in the case of large doses.

Danger, therefore, lies in high percentages, absolute safety in low percentages.

